# Controllable Synthesis of Monodisperse CeO_2_ Nanoparticles with Tunable Sizes for Chemical-Mechanical Polishing

**DOI:** 10.1021/acsomega.5c02209

**Published:** 2025-08-06

**Authors:** Yange Ma, Tuquan Qiu, Zhenxiang Zhao, Quan Zou, Bo Chen, Qian Sun, Shuangliang Zhao

**Affiliations:** † State Key Laboratory of Featured Metal Materials and Life-cycle Safety for Composite Structures and School of Chemistry and Chemical Engineering, 12664 Guangxi University, Nanning 530004, P. R. China; ‡ Guangxi Key Laboratory of Petrochemical Resource Processing and Process Intensification Technology, Guangxi University, Nanning 530004, P. R. China

## Abstract

CeO_2_ has been widely employed in semiconductors and ultraprecision optical polishing on account of its remarkable physical, chemical, and mechanical qualities. In this work, we present an efficient strategy to prepare monodisperse CeO_2_ nanoparticles (NPs) with tunable sizes through a solvothermal method integrated with the following in situ surface modification procedure. The as-prepared CeO_2_ NPs exhibit excellent monodispersibility and regular spherical morphology. Their average particle diameter can be readily regulated from 7.0 to 336.3 nm by adjusting the experimental conditions. Subsequently, these obtained CeO_2_ NPs can be formulated into different slurries, and their polishing performances are tested. According to the results, the CeO_2_ polishing slurry with an average particle size of 94.5 nm has a material removal rate of 481 nm/min and a surface roughness of 0.14 nm. In contrast, the commercial product has only a material removal rate of 214 nm/min and a surface roughness of 0.30 nm. Obviously, a much faster polishing rate and a smoother surface can be achieved. Furthermore, a smaller surface roughness of 0.11 nm can be realized when the average particle size of CeO_2_ is reduced to 46.9 nm, which is beneficial to the ultraprecision optical polishing process and can greatly improve the quality of the material surface.

## Graphical abstract

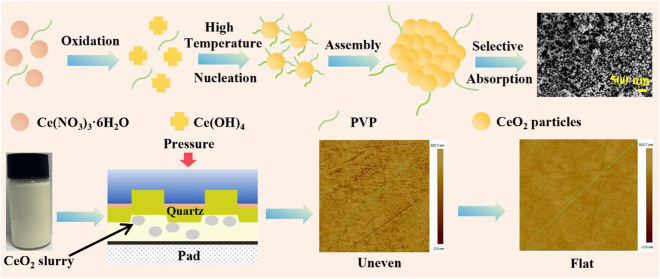

## 1 Introduction

Chemical-mechanical polishing (CMP) is a technique that integrates chemical and mechanical actions to achieve uniform surface planarization and superior surface quality of materials, which is widely used in the production of semiconductors and ultraprecision optical components.^1–3^ The polishing performance of CMP depends on various factors, including the composition of the polishing slurry, operational conditions, and the characteristics of the polishing pads.^4,5^ Among these factors, the polishing slurry is the key element that affects the performance of CMP. The size and morphology of abrasive particles, as the primary constituent of polishing slurry, have a direct impact on both material surface quality and polishing efficiency.^6–10^

CeO_2_ is widely recognized as a critical polishing material due to its exceptional overall performance. The proper hardness promotes good intimate contact with the polished materials, thereby reducing the potential for surface damage in CMP applications. In addition, exceptional chemical stability of CeO_2_ particles enables it to retain stable over extended periods in diverse chemical environments without significant degradation or transformation, which possesses remarkable redox properties, enabling rapid switching between oxidized and reduced states.^11,12^ As a kind of eco-friendly materials, CeO_2_ also complies with the environmental standards of contemporary industries, thus being extensively employed in CMP.^13–16^ Moreover, their high-quality polishing capability ensures rapid task completion, boosting productivity and achieving a flat and smooth surface.^17–20^

During the development of CeO_2_ abrasive particles, there are some challenges in achieving precise control of particle size, primarily due to irregular morphology and poor dispersibility, which may significantly impair the optical and mechanical properties of CeO_2_.^21,22^ For instance, Gnanam and Rajendran^23^ employed a straightforward sol–gel approach to fabricate the Cd-doped CeO_2_ nanoparticles, achieving well-dispersed particles with uniform spherical/elliptical morphology. However, this method suffered from the problems of a relatively long reaction period and rather high cost, and these ion-doped particles were not easy to be cleaned after polishing, which may cause contamination problems to the environment. Oh et al.^24^ prepared the CeO_2_ particles of diverse sizes and shapes with good dispersibility by a hydrothermal method, but the shapes of particles were not stable, which resulted in a large difference of the polished surface roughness. Eka Putri et al.^25^ developed an environmentally friendly precipitation approach to prepare spherical CeO_2_ nanoparticles with high purity. Nevertheless, their particle size was relatively small and hindered effective embedding into polished material surfaces, leading to a decline in surface quality. Therefore, a simple and green preparation approach of monodisperse CeO_2_ NPs with uniform and controllable size and morphology is still a major and meaningful task.^26,27^

In this study, we conduct a series of experiments to synthesize monodisperse CeO_2_ NPs with tunable sizes and regular spherical morphology employing a solvothermal procedure in combination with an in situ surface modification treatment. The effects of various reaction conditions, such as solvothermal temperature, time, and concentration of cerium salt solution were investigated. Furthermore, the polishing properties of the as-prepared CeO_2_ NPs have also been evaluated in terms of both the material removal rate (MRR) and the surface quality, after the CeO_2_ NPs have been formulated into slurries and compared to those of a commercial product.

## 2 Experimental Section

### 2.1 Materials

Cerium nitrate hexahydrate (Ce­(NO_3_)_3_·6H_2_O) and polyvinylpyrrolidone (PVP) were supplied by Sinopharm Chemical Reagent Co., Ltd. Commercial CeO_2_ was sourced from Zhuhai Lizhiyi New Material Co., Ltd. Ethanol (C_2_H_5_OH, ≥ 99.7%) and ethylene glycol (C_2_H_6_O_2_, 98%) were purchased from Sichuan Xilong Science Co., Ltd. and McLean Biochemical Technology Co., Ltd., respectively. The dispersant Byk-154 was purchased from Dongguan Shiyu New Materials Co., Ltd. Sodium hexametaphosphate (SHMP, AR) was supplied by Guangxi Liuzhou Yixuan Chemical Co., Ltd. Sodium polyacrylate (SAP, AR) was purchased from Chengdu Kelong Chemical Products Co., Ltd. Triethanolamine (TEA, AR) was supplied by Tianjin Fuyu Fine Chemical Co., Ltd. Deionized water was prepared in the laboratory.

### 2.2 Preparation of CeO_2_ Nanodispersion

Figure 1 illustrates the synthesis process of the CeO_2_ nanodispersion. A solvothermal method combined with a surface modification technology was utilized to prepare CeO_2_ NPs. Initially, Ce­(NO_3_)_3_·6H_2_O (5 g) and PVP (2 g) were dissolved in a mixed solvent of ethylene glycol and deionized water (volume ratio 15:1). The above solution was stirred magnetically until it became clear and transparent. Subsequently, the mixture solution was transferred into a 150 mL PTFE-lined autoclave and heated at 140 °C for 8 h. After cooling to room temperature, the product was centrifuged at 11000 rpm for 10 min and washed with deionized water and ethanol for three times. The product was dispersed in deionized water and ultrasonicated at 400 W for 10 min to achieve a CeO_2_ nanodispersion. By variation of the reaction duration (4–12 h), reaction temperature (120–170 °C), and cerium salt concentration (0.03–0.60 mol/L), CeO_2_ NPs with different particle sizes were successfully synthesized.

**Figure 1:**
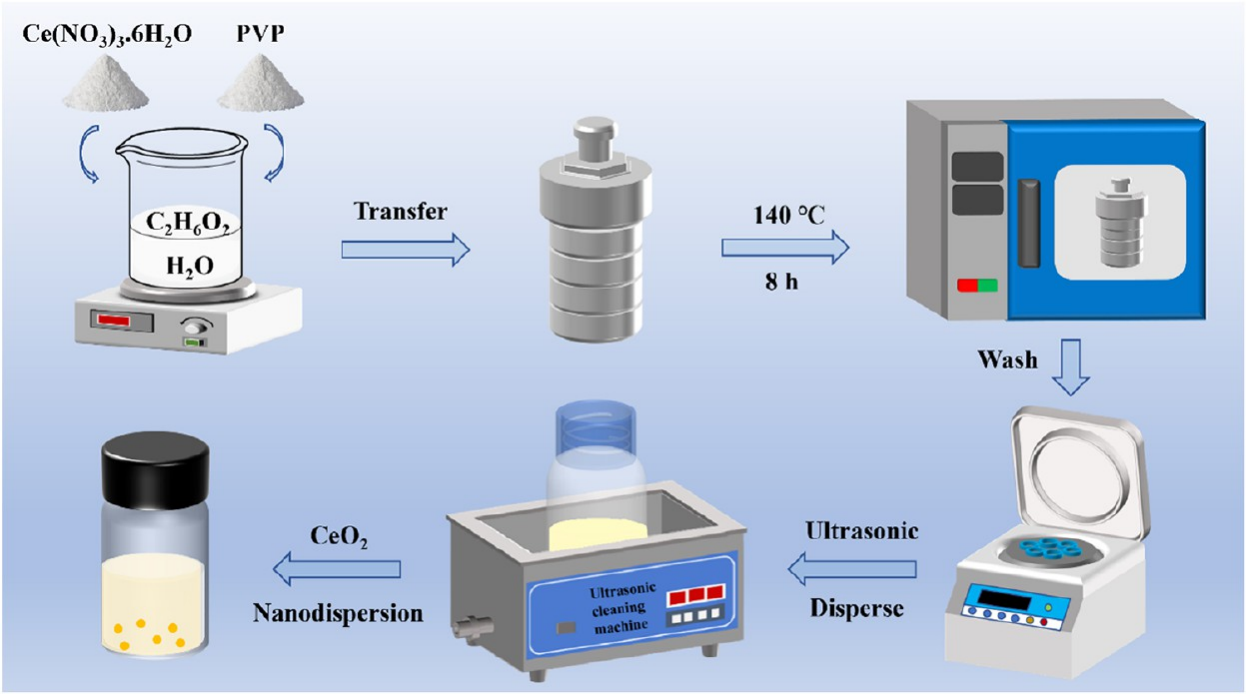
Schematic diagram of the synthesis of CeO_2_ nanodispersion.

### 2.3 Characterization

The particle size, morphology, and dispersibility of CeO_2_ NPs were analyzed by using a transmission electron microscope (TEM, F200, JEOL, Japan) and a scanning electron microscope (SEM, Sigma 300, ZEISS, Germany). Nano Measurer software was used to measure the particle size distributions based on the TEM images. The crystal structure was characterized by X-ray diffraction (XRD, A24A10, Bruker, Germany) at 40 kV and 40 mA. Fourier Transform Infrared (FTIR) spectra were acquired with a Nicolet iN10 spectrometer (Thermo Fisher Scientific) in the range of 4000–400 cm^–1^. Surface composition was analyzed by X-ray photoelectron spectroscopy (XPS, K-α, Thermo Scientific). Thermal behavior was evaluated using a thermogravimetric analysis (TGA) system (TA TGA55) under nitrogen at a heating rate of 10 °C/min. The surface morphology of sample was characterized using atomic force microscopy (AFM) analysis system (Dimension Icon, Bruker Germany).

### 2.4 Polishing Experiments

The commercial and synthesized CeO_2_ NPs were adjusted to 10 wt % solid content at room temperature, then sonicated, and mixed with the following reagents, respectively, to formulate different polishing slurries. Table 1 outlines the reagents needed for slurry preparation, specifying the mass of each reagent per kilogram of dispersant (g/kg).

**Table 1:** Required Reagents of Slurry and Their Related Requirements

reagents	dosage (g/kg)	other requirements
Byk-154	70	42% nonvolatile
SHMP	3	
SAP	120	average molecular weight 3000–5000 w
TEA	50	

The polishing slurries described above were ultrasonically dispersed. The quartz glass (JGS1 quartz) and polishing pads (Beili MLK66) were purchased from Zhuhai Lizhiyi New Material Co., Ltd. The standard size of the quartz was 40 mm in diameter and 2.6 mm in thickness. The polishing machine used for the CMP experiments was a Digiprep 251 model (Metkon). In addition, semiconductor polishing samples underwent processing at 10 N pressure, with head and disk speeds set to 100 and 240 rpm, for a duration of 20 min. The polishing equipment diagram will be shown together with the mechanism diagram in Figure 6a in Section 3.2.

For ultraprecision optical polishing, the parameters were set to a pressure of 8 N, 60 rpm head speed, and 120 rpm disk speed for 20 min. After being polished, the JGS1 quartz samples were cleaned by ultrasonic washing with deionized water and then dried in air. Their surface morphology was analyzed using AFM in noncontact mode over a 5 μm × 5 μm scanning area. The mass of the quartz samples before and after polishing was measured, and the material removal rate (MRR) was calculated by using the provided equation^21^

1 $$\mathrm{MRR}=\frac{\Delta m}{\rho .S.t}$$

Where ρ is the density of JGS1 quartz (2.2 g/cm^3^), *S* is the area of the polished surface (12.6 cm^2^), and *t* is the polishing time (20 min). The values of MRR and surface roughness (Ra) were determined by averaging the results for three repeated experiments.

## 3 Results and Discussion

### 3.1 Preparation of CeO_2_ NPs with Tunable Particle Sizes

We synthesized CeO_2_ NPs with varying particle sizes and selected PVP-modified CeO_2_ for a series of characterizations. The modified CeO_2_ was prepared under the conditions of a 140 °C reaction temperature, 8 h reaction time, and 0.06 mol/L cerium salt solution concentration. Figure 2a shows an XRD comparison of commercial CeO_2_ NPs and PVP-modified CeO_2_ NPs. The XRD pattern of modified CeO_2_ NPs exhibits stronger and sharper diffraction peaks, indicating higher crystallinity, compared with commercial CeO_2_ NPs. The distinct peaks can be observed at 2θ = 28.7, 33, 47.5, and 56.5°, which correspond to the (111), (200), (220), and (311) crystal planes, respectively. The peak positions and intensities align well with the CeO_2_ standard map (JCPDS Card No: 34-0394),^1^ and the absence of impurity peaks confirms the formation of pure CeO_2_ with a cubic fluorite structure.

**Figure 2:**
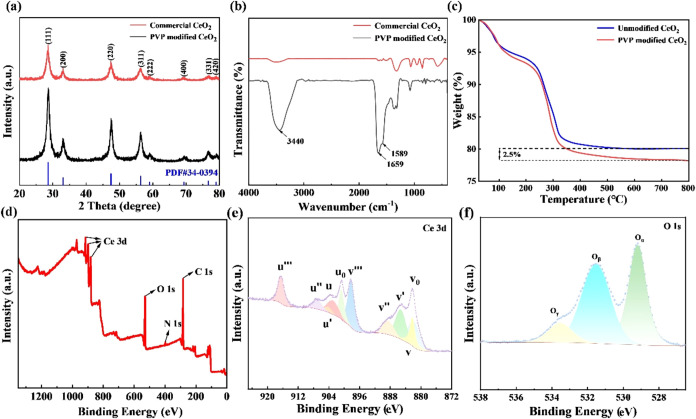
(a) XRD patterns and (b) FTIR spectra of commercial and PVP-modified CeO_2_ NPs, (c) TG curves of PVP-modified CeO_2_ NPs and unmodified CeO_2_ NPs, (d) XPS spectra, (e) Ce 3d XPS spectra, and (f) O 1s XPS spectra of PVP-modified CeO_2_ NPs.

Figure 2b presents the FTIR spectra of commercial CeO_2_ NPs and PVP-modified CeO_2_ NPs. The peak at 3440 cm^–1^ corresponds to the stretching vibration of the OH^–^ group, indicating the presence of free water in the product. The 1659 and 1589 cm^–1^ peaks result from the vibrations of the C–N and C=O groups, respectively, demonstrating coordination with the −NH-C=O group of PVP during the reaction process.^15^ Figure 2c shows the TG curves of pure CeO_2_ NPs and PVP-modified CeO_2_ NPs. The PVP-modified CeO_2_ NPs exhibits two distinct weight loss stages. The initial weight loss below 200 °C is primarily due to the decomposition of surface-adsorbed crystalline water, while the subsequent weight loss indicates the onset of PVP decomposition on the surface of CeO_2_ NPs. The TG results reveal a PVP grafting rate of 2.5% on the spherical CeO_2_ NPs.

Figure 2d illustrates the XPS spectrum of PVP-modified CeO_2_ NPs. A peak at 399.1 eV, corresponding to the N 1s binding energy, demonstrates the existence of Ce, O, and C in PVP-modified CeO_2_ NPs. The Ce 3d spectrum is resolved into u and v components for Ce 3d_3/2_ and 3d_5/2_. As depicted in Figure 2e, the u‴, u″, u, v‴, v″, and v peaks demonstrate Ce^4+^ signatures, while the u′, u_0_, v′, and v_0_ features explicitly confirm the presence of Ce^3+^.^28,29^ The Ce^3+^ and Ce^4+^ peaks are identified using a nonlinear Gaussian peak fitting method after background subtraction. In CMP, Ce^3+^ on the surface of CeO_2_ slurries can serve as catalytic sites, facilitating tribochemical interactions between silica particles of quartz glass and ceria components,^30^ thereby enhancing the MRR for silica. In Figure 2f, additionally, the O 1s XPS spectrum exhibits three peaks at approximately 529.2, 531.6, and 533.5 eV, corresponding to bulk lattice oxygen (O_α_), surface oxygen related to adsorbed ions or oxide defects (O_β_), and oxygen derived from surface-adsorbed water or carbonate moieties (O_γ_).^13,31^

Figure 3a depicts the mechanism of CeO_2_ particle formation. Ce^3+^ is first oxidized to Ce^4+^ by NO_3_^–^, followed by the reaction of Ce^4+^ with OH^–^ to produce Ce­(OH)_4_. Under the appropriate temperature and pressure conditions in the reactor, Ce­(OH)_4_ nucleates and dehydrates to form CeO_2_ grains. These grains then self-assemble under the influence of surface tension. The adsorption of PVP on the grain surfaces slows the assembly process, favoring the formation of quasispherical CeO_2_ particles. The PVP molecules play a critical role in regulating the nucleation and growth kinetics by selectively adsorbing on high-energy crystal facets, which promotes isotropic growth while suppressing particle aggregation through steric stabilization. With further reaction, the crystallinity increases, and the selective adsorption of C_2_H_6_O_2_ and PVP drives the final formation of spherical CeO_2_ particles.^32,33^ Figure 3b–g displays the TEM images and the associated size distribution of CeO_2_ NPs prepared for varying reaction durations. The CeO_2_ NPs maintain monodispersity throughout the process and display a uniform spherical morphology. As the reaction time increases from 4 to 12 h, the average particle size grows from 7.0 to 133.8 nm, due to Ostwald ripening,^15^ which validates that the reaction time plays a crucial role in controlling the particle size of CeO_2_ NPs during the preparation process.

**Figure 3:**
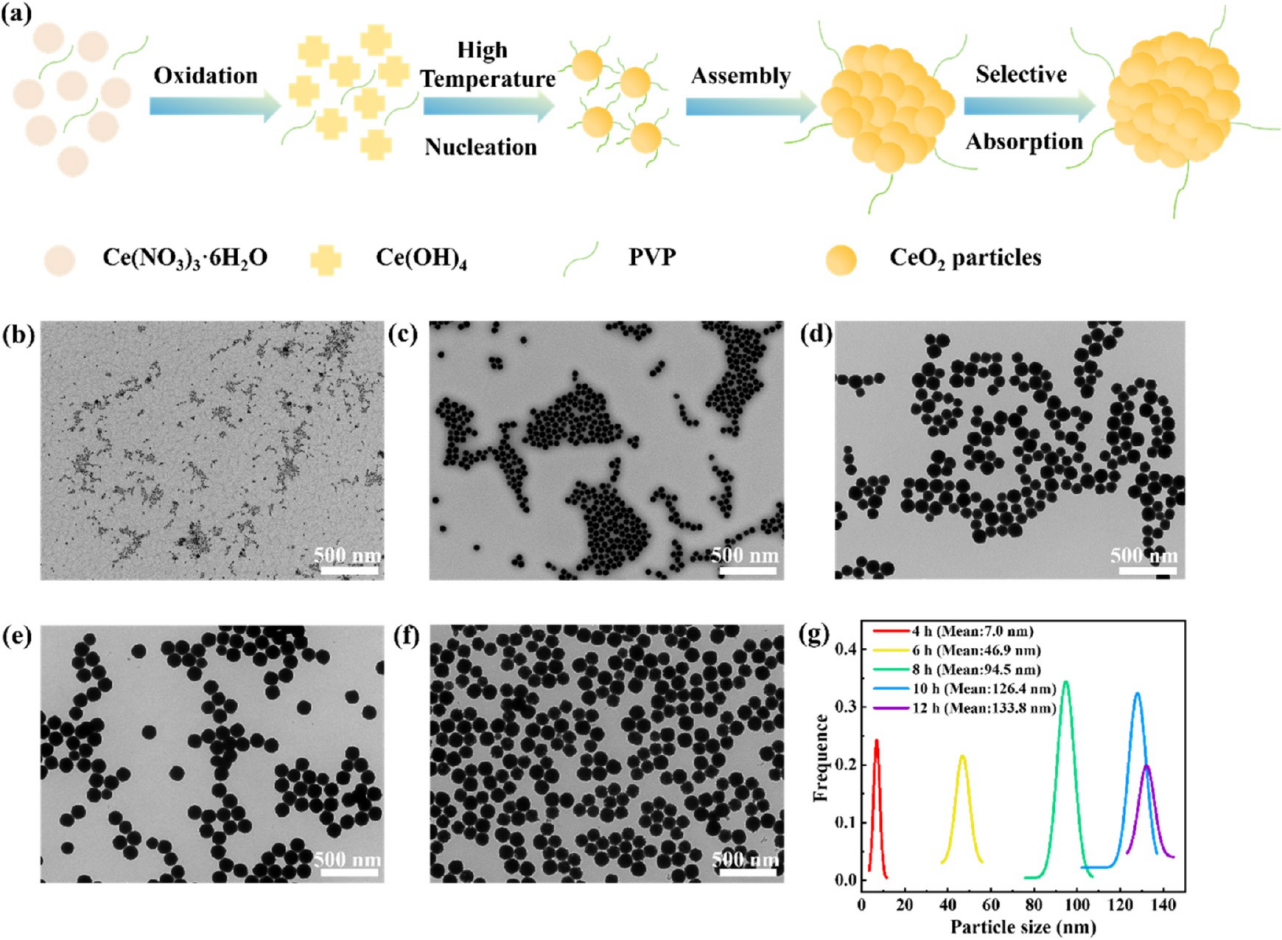
(a) Synthesis mechanisms of PVP-modified CeO_2_ NPs, TEM images of CeO_2_ NPs prepared for different reaction durations: (b) 4, (c) 6, (d) 8, (e) 10, and (f) 12 h and (g) their corresponding particle size distribution.

Figure 4 presents the morphology of PVP-modified CeO_2_ NPs along with their corresponding particle size distributions at different temperatures. The results show that most CeO_2_ NPs display a monodisperse and regular spherical morphology throughout the temperature increase process. In Figure 4a, spherical particles with a size of 26.5 nm begin to form at 120 °C. Figure 4b–d demonstrates that as the temperature increases from 130 to 150 °C, the particle size shows a trend of initially decreasing and then increasing, and the reason is likely Ostwald Ripening.^34^ During the whole heating process, small particles gradually dissolve and redeposit onto large particles. In Figure 4d, when the temperature reaches 150 °C, the particle size attains a maximum value of 279.8 nm. As shown in Figure 4e,f, with a further increase in the temperature, the particles gradually become more uniformly distributed. The elevated temperature drives the removal of moisture, thereby reducing interfacial energy and causing the particle size to decrease accordingly.

**Figure 4:**
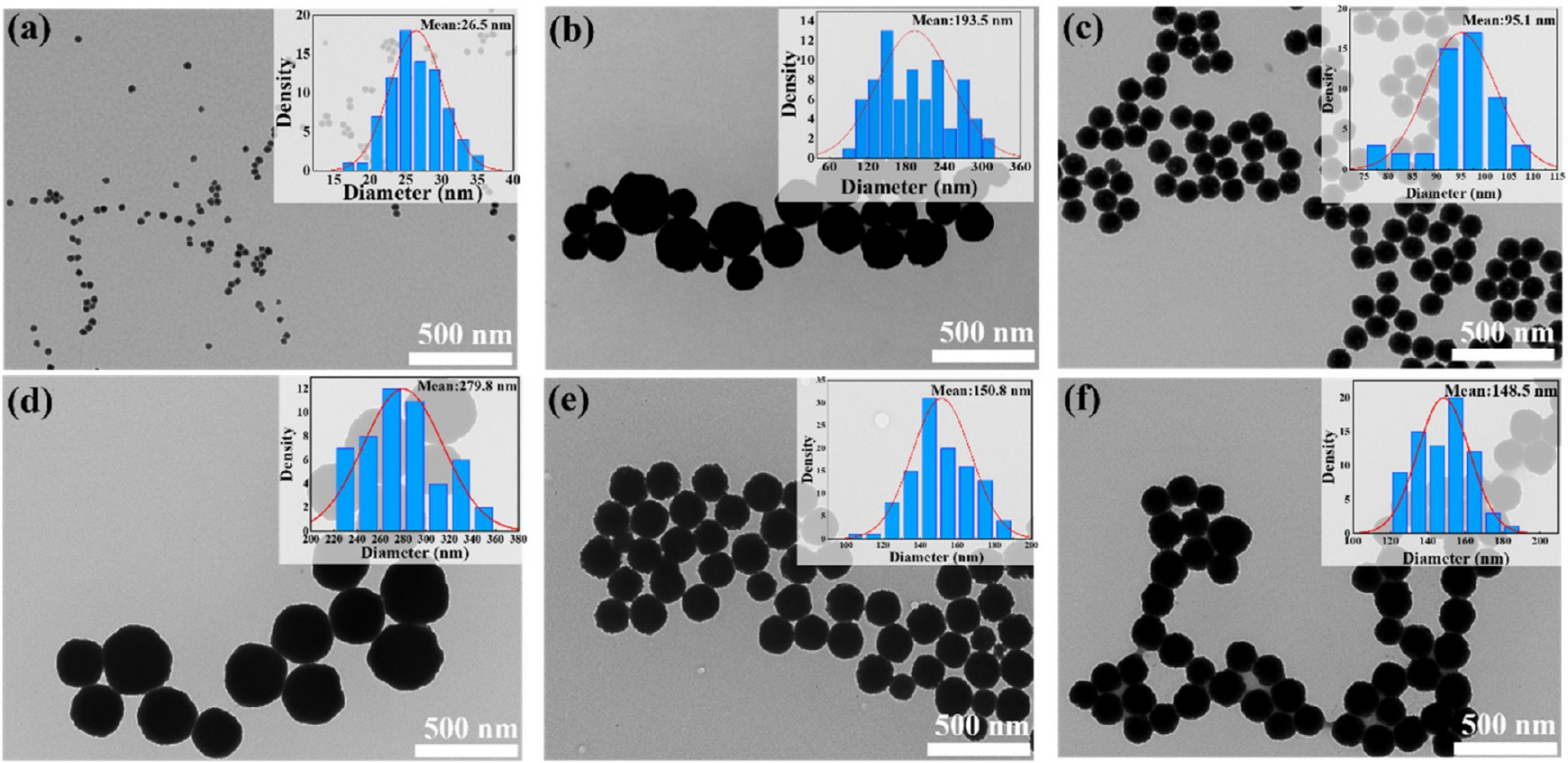
TEM images and size distribution of CeO_2_ NPs prepared at different reaction temperatures: (a) 120, (b) 130, (c) 140, (d) 150, (e) 160, and (f) 170 °C.

Figure 5 shows TEM images of CeO_2_ NPs prepared at different cerium salt concentrations. Under the premise of ensuring the high dispersibility of CeO_2_ NPs, experiments with increasing cerium salt concentration were conducted. As shown in Figure 5, the influence of the cerium salt concentration on the morphology and size distribution of CeO_2_ NPs is revealed through TEM analysis. As seen, the CeO_2_ NPs are monodisperse throughout the process, and their morphology shows a regular spherical shape.

**Figure 5:**
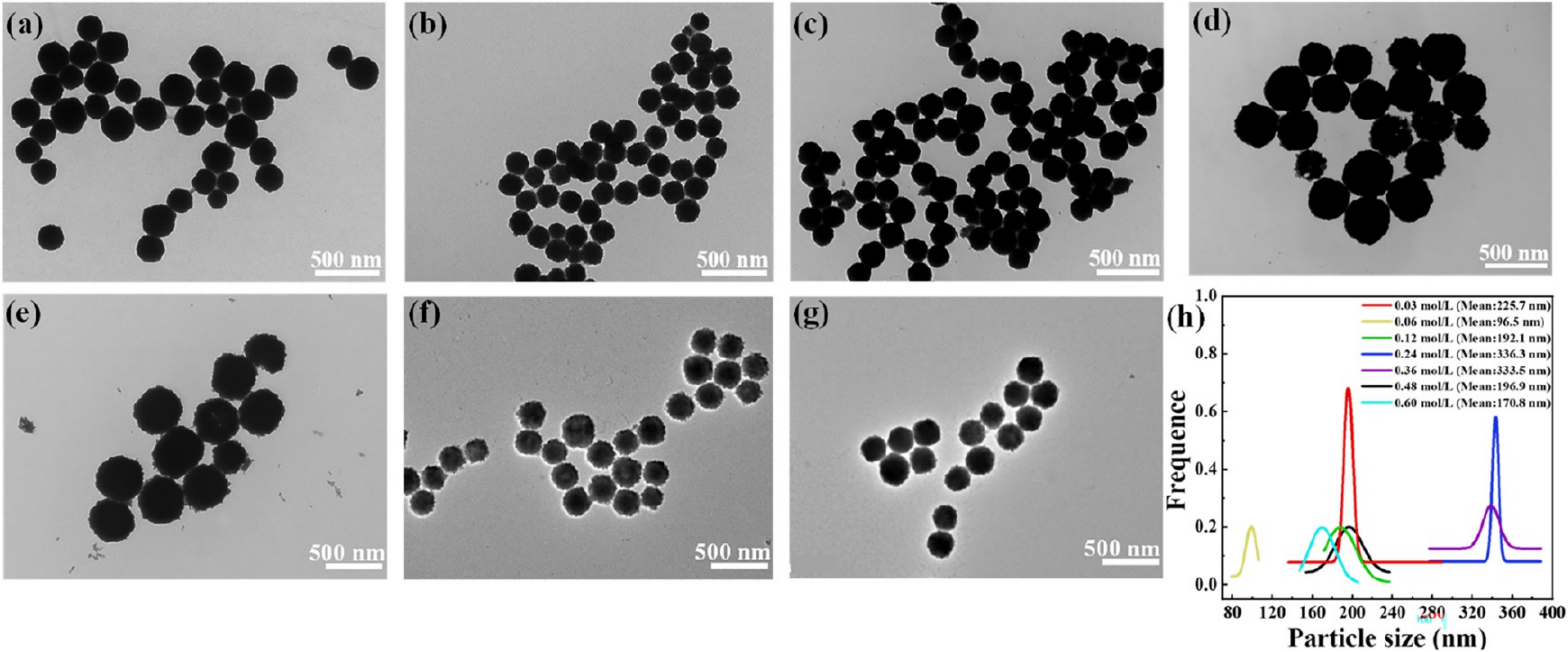
TEM images of modified CeO_2_ NPs prepared using different cerium salt concentrations: (a) 0.03, (b) 0.06, (c) 0.12, (d) 0.24, (e) 0.36, (f) 0.48, and (g) 0.60 mol/L and (h) their corresponding particle size distribution.

Figure 5a indicates that at a cerium salt concentration of 0.03 mol/L, the imbalance between nucleation and particle growth causes a limited number of nuclei to form, which are dispersed at low concentrations. Meanwhile, the growth process is influenced by reactant diffusion and the local chemical environment, resulting in inconsistent growth rates among the nuclei. Consequently, the particles with nonuniform sizes are produced. Figure 5b–g demonstrates that as the cerium salt concentration increases, the average particle size follows a trend of decreasing-increasing-decreasing. This behavior can be explained by the gradual intensification of agglomeration with higher cerium salt concentrations, leading to an initial increase in the particle size. However, when the concentration increases further, the solution reaches a supersaturated state. At this stage, a critical balance is achieved between agglomeration and dispersion, causing the particle size to decrease once again.^32^

### 3.2 Polishing Mechanism of the Prepared CeO_2_ Slurries

Figure 6 shows the polishing mechanism of the CeO_2_ polishing slurries. Figure 6a illustrates the experimental setup for polishing. The removal of excess material is accomplished through tribochemical reactions at the quartz-slurry interface.^35,36^ The monodisperse CeO_2_ NPs enhance pore infiltration of the polishing solution, enabling direct contact between the workpiece and the oxidized components of the solution.^37–39^ A hydrothermally softened layer forms on the quartz surface, where the uniform-sized CeO_2_ NPs promote the generation of localized shear stress fields, thereby enhancing their removal efficiency. Subsequently, this softened layer is removed through frictional interactions between the slurry particles and the layer itself. Simultaneously, the used slurry particles involved in the polishing process and the materials stripped from the quartz surface are washed away by the polishing action.^40–42^ This cyclic process continues until the entire workpiece surface achieves uniform smoothness, resulting in global planarization.^43^

**Figure 6:**
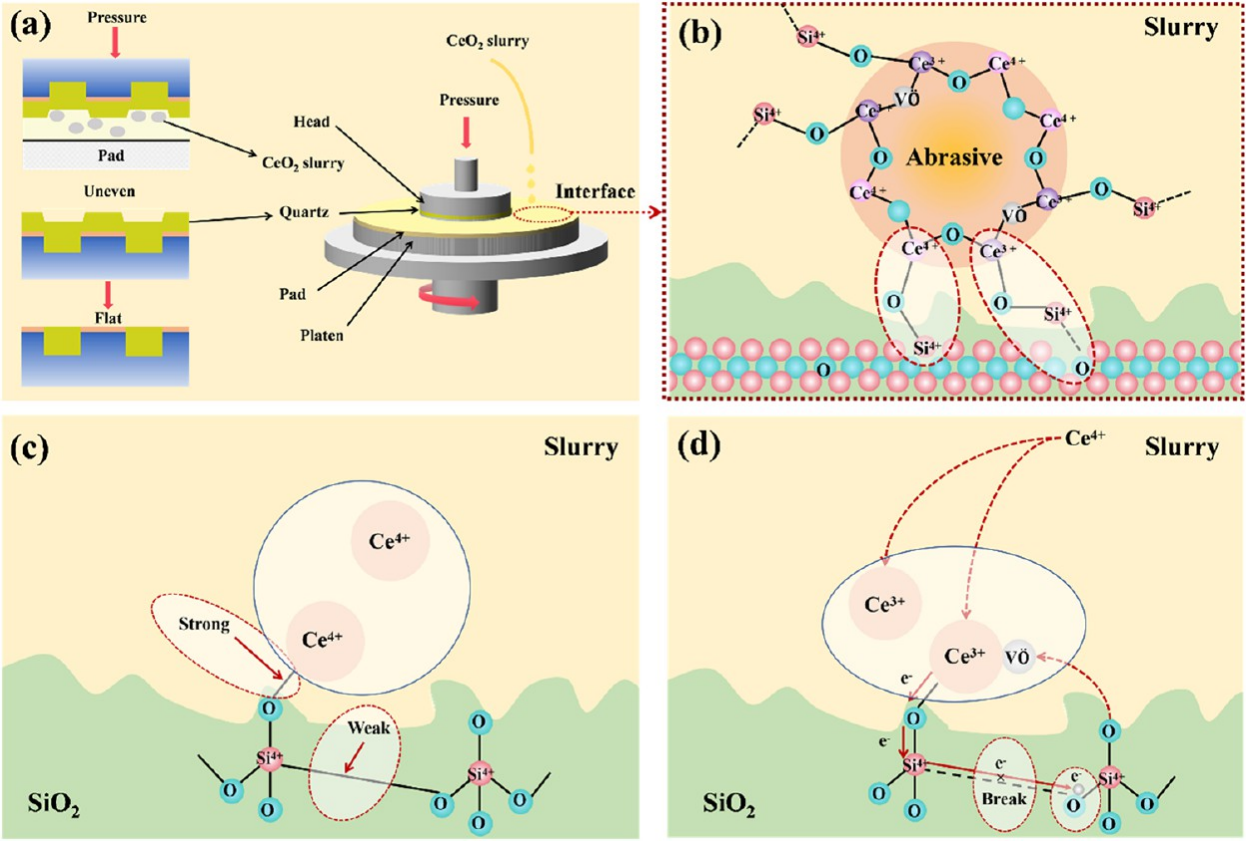
(a) Schematic depiction of the polishing experimental setup, (b) model for the removal process of CeO_2_ slurries, and (c, d) reaction model between Ce^4+^/Ce^3+^ and the SiO_2_ substrate.

Figure 6b depicts the mechanism involved in the removal of the CeO_2_ slurry from SiO_2_ surfaces. This mechanism utilizes the chemical tooth concept that was advanced by Cook^44^ as a foundation, and bonding interactions transpire at the interface. Following hydrolysis, the SiO_2_ surface terminates with Si–OH groups, while Ce–OH groups form when the CeO_2_ slurries are dispersed in water. These groups interact with SiO_2_ surface sites, speeding up the formation of Ce–O–Si bonds, as detailed in eqs 2–4. Within CeO_2_ crystals, the Ce element primarily adopts two valence states: Ce^3+^ and Ce^4+^, each playing a distinct role in the SiO_2_ removal process.

Figure 6c depicts Ce^4+^-mediated bond weakening. Robust Ce–O–Si linkages induce tensile stress on adjacent Si–O–Si networks, with homogenized nanoparticle size distribution minimizing stress concentration points. Then the mechanical force application then cleaves the destabilized Si–O bonds. Meanwhile, Figure 6d illustrates the reactive model involving Ce^3+^ and the SiO_2_ substrate. Ma et al.^19^ have shown that Ce^3+^ forms as a result of Ce^4+^ combining with free electrons, which are produced during the formation of oxygen vacancies. After Ce–O–Si bonds are created, the free electrons present in Ce^3+^ migrate toward the SiO_2_ surface, further weakening or even disrupting the stable Si–O covalent bond. The agglomeration of CeO_2_ NPs is suppressed, increasing the presence of Ce^3+^, and the presence of Ce^3+^ within the slurry significantly enhances the efficiency of breaking Si–O bonds, thereby enabling a higher polishing rate.

2 $$\begin{array}{l} \equiv \mathrm{Si}-O-\mathrm{Si}\equiv +H-\mathrm{OH}↔\;\equiv \mathrm{Si}-\mathrm{OH}+\mathrm{HO}-\mathrm{Si}\equiv \end{array}$$

3 $$\begin{array}{l} =\mathrm{Ce}-O-\mathrm{Ce}=+H_{2}O↔\;=\mathrm{Ce}-\mathrm{OH}+\mathrm{HO}-\mathrm{Ce}= \end{array}$$

4 $$\begin{array}{l} =\mathrm{Ce}-\mathrm{OH}+\mathrm{HO}-\mathrm{Si}↔\;=\mathrm{Ce}-O-\mathrm{Si}\equiv +\;H_{2}O \end{array}$$

### 3.3 CMP Performances of CeO_2_ Slurries

#### 3.3.1 Surface Analysis for Semiconductor Quartz Glass Polishing

Figure 7a,b shows the SEM images of commercial CeO_2_ NPs and our synthesized CeO_2_ NPs prepared under the conditions of 140 °C reaction temperature, 8 h reaction time, and 0.06 mol/L cerium salt solution concentration. The average particle size of our synthesized CeO_2_ NPs is 94.5 nm as shown in Figure 3d. Compared with the commercial CeO_2_ NPs, our prepared CeO_2_ NPs exhibit a more regular spherical morphology and higher dispersibility. Figure S1e displays the Zeta potential and storage stability of the commercial CeO_2_ slurries and our prepared CeO_2_ slurries. It can be observed that our CeO_2_ slurries have a relatively high surface potential, which enables the CeO_2_ NPs to be uniformly dispersed in aqueous medium. Furthermore, the commercial CeO_2_ slurries begin to separate after 10 days, while our CeO_2_ slurries remain in a suspended state. Figure 7c shows the corresponding EDS spectra, indicating that the C, O, Ce, and N elements are uniformly distributed on the surfaces of CeO_2_ NPs.

**Figure 7:**
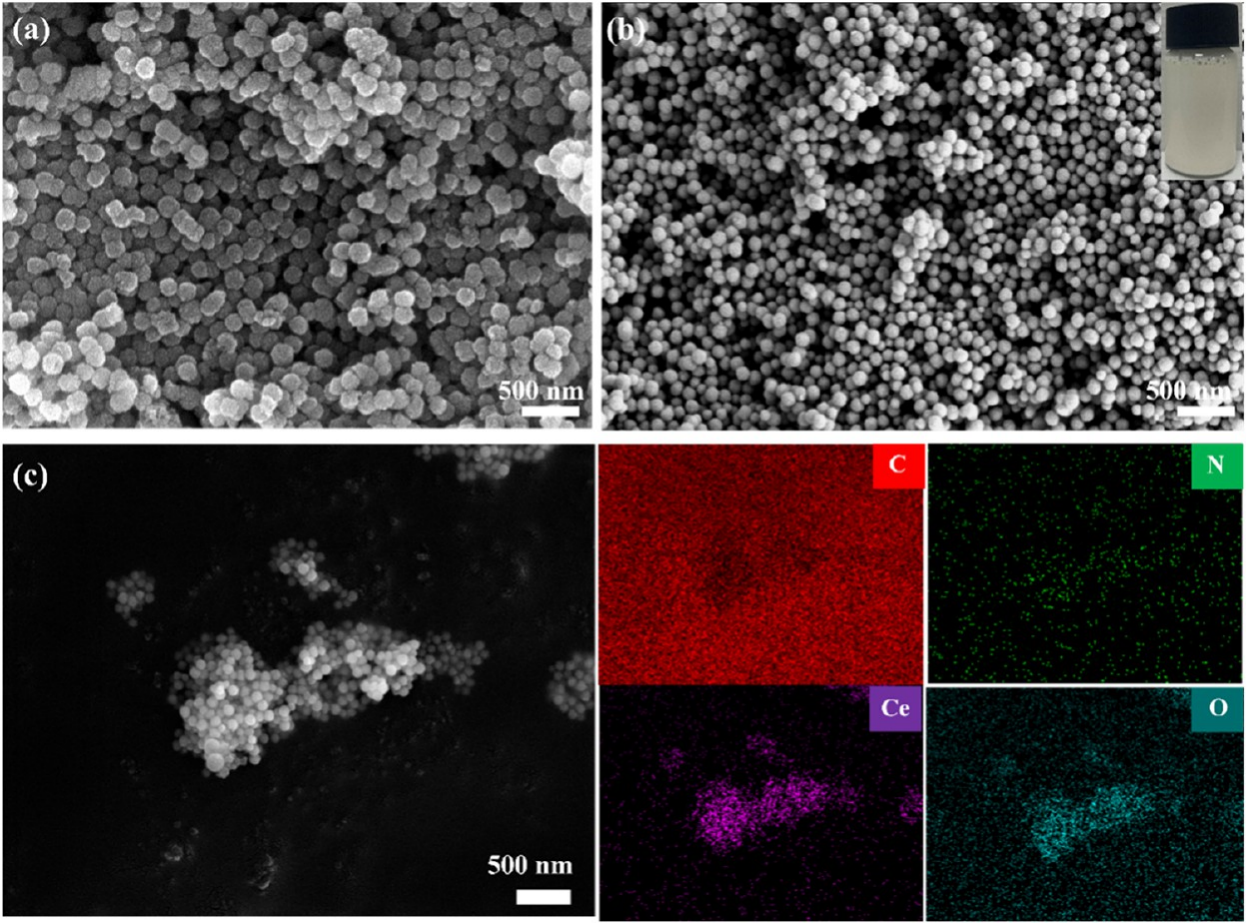
(a) SEM image of commercial CeO_2_ NPs, (b) SEM image and digital photograph, and (c) EDS image of our modified CeO_2_ NPs.

The surface characteristics of polished samples are critical parameters for assessing slurry polishing performance. Figure 8a–c displays the two-dimensional (2D) AFM images of the quartz surface before and after polishing, and Figure 8d–f presents the corresponding line-scan profiles. Figure 8a shows the initial surface morphology of the unpolished JGS1 quartz glass. It can be seen that the surface of the quartz glass before polishing has a remarkable difference in color distribution and significant scratches. For a more intuitive comparison, we choose the diagonal of the 2D-AFM image to measure the surface contour roughness, where the *X*-axis represents the length of the line profile and the *Z*-axis its depth, and the improvement in surface roughness is demonstrated by the smoother curve. The curve in Figure 8d exhibits significant fluctuations, indicating poor surface flatness, with a peak value of 3.26 nm and a valley value of 1.19 nm along the measurement direction.

**Figure 8:**
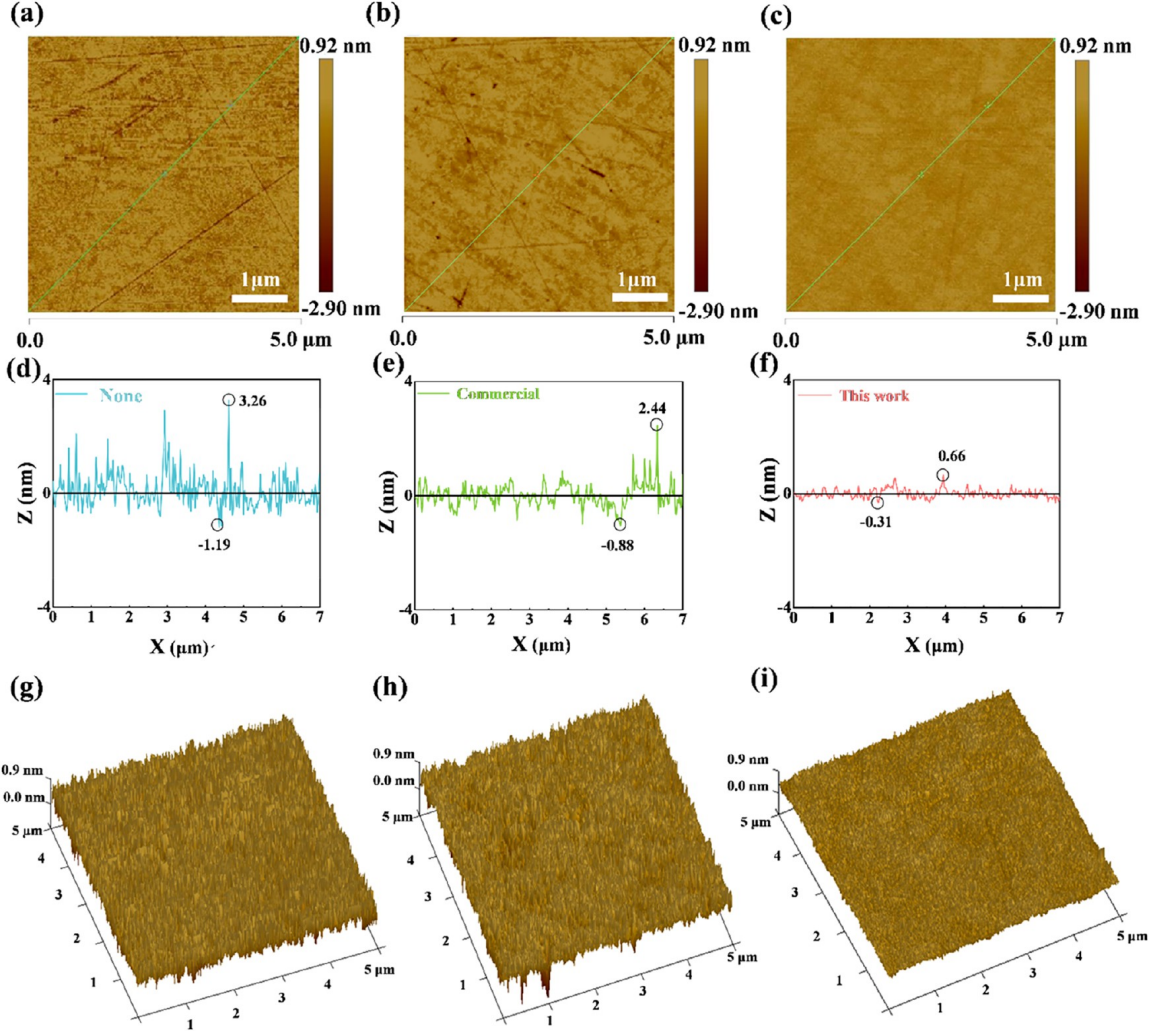
2D-AFM images of the surfaces (a) before CMP and after CMP using (b) commercial slurry, (c) our slurry, (d–f) the corresponding line-scan profiles of their surface, and (g–i) their corresponding 3D-AFM images.

Figure 8b shows the surface morphology after polishing with the commercial slurry. It can be observed that the scratches are slightly reduced compared to those before polishing, but a significant number of scratches still remain. The corresponding curve in Figure 8e shows peaks of 2.44 nm and valleys of 0.88 nm along the measurement direction, exhibiting slightly reduced fluctuations compared to the prepolished state. Figure 8c displays the polished surface morphology using the slurry prepared from our CeO_2_ samples, which are prepared at 140 °C reaction temperature, 8 h reaction time, and 0.06 mol/L cerium salt solution concentration. There are few scratches on the surface of the quartz glass in comparison with the previous two, and this surface possesses remarkable flatness and homogeneity, suggesting a perfect improvement in the overall surface quality. Compared to the line-scan profile curves for other conditions, the curve in Figure 8f has gentler undulations, indicating improved surface flattening and lower roughness, with a peak value of 0.66 nm and a trough value of 0.31 nm along the measurement direction.

To demonstrate quartz surface morphology changes before and after CMP, three-dimensional atomic force microscopy reconstructions were created from two-dimensional scans through NanoScope Analysis 2.0. As seen in Figure 8g, many nanoscale bumps are visible on the surface before the CMP. In Figure 8h, after the commercial slurry is used, surface unevenness begins to decrease. Figure 8i shows that after applying the slurries from our study, most of the nanoscale bumps are removed, resulting in a highly flat surface without any new mechanical or chemical defects, ultimately achieving a high-quality ultrasmooth surface.

#### 3.3.2 Removal Efficiency for Semiconductor Quartz Glass Polishing

MRR serves as a crucial measure of the effectiveness of material polishing processes. Figure 9a shows the average MRR data obtained from the CMP of JGS1 quartz polished parts with a commercial CeO_2_ slurry and our CeO_2_ slurry at the same concentration, respectively. All parameters remain consistent throughout the CMP. Data can be calculated in eq 1. As can be seen from Figure 9a, the average MRR of the CeO_2_ slurry in this study is 481 ± 0.41 nm/min and that of the commercial CeO_2_ slurry is 214 ± 0.21 nm/min. Our CeO_2_ slurry shows an improvement of about 125% over the commercial CeO_2_ slurry and thus provides a higher polishing efficiency. Figure 9b summarizes the surface roughness in the three cases of nonpolishing, using commercial slurry and our slurry. It can be found that the Ra of the quartz glass before polishing is 0.47 ± 0.0049 nm and the Rq is 0.66 ± 0.0056 nm, which is not much different from the surface of the quartz glass after polishing by the commercial slurry (Ra is 0.30 ± 0.0034 nm and Rq is 0.42 ± 0.0031 nm). In contrast, the present study shows that the quartz glass after slurry polishing has a Ra of 0.14 ± 0.0017 nm and Rq of 0.18 ± 0.0022 nm, which has a lower surface roughness.

**Figure 9:**
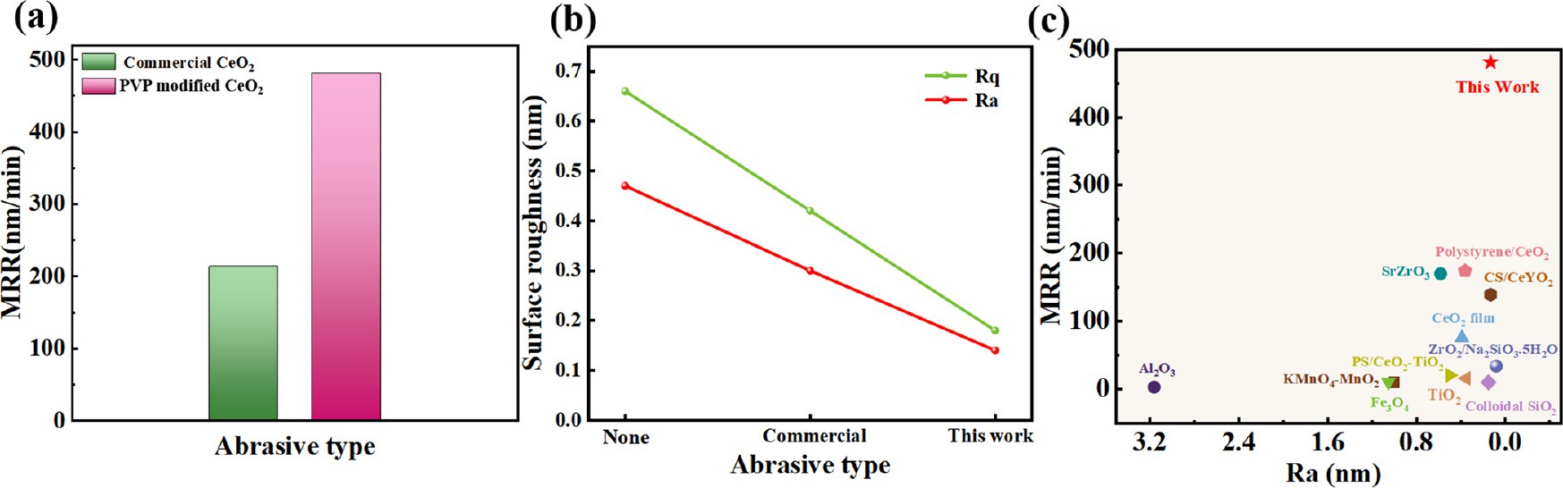
(a) MRR polished under CMP by the different slurries with the same concentration, (b) glass surface roughness results before and after polishing, (c) efficiency of CMP reported in the literature and herein.^1,2,45–52^

The two key metrics, MRR and Ra, demonstrate that the slurry developed in this study significantly enhances both material surface quality and removal efficiency compared to commercial slurries, showing excellent polishing performance. This improvement can be attributed to the high dispersibility and sphericity of the modified CeO_2_ NPs. Figure 9c compiles MRR and Ra data from a wide range of reported works using different polishing materials.^2,45–49^ By comparison of these data with the results of the present study, it is evident that our slurries exhibit superior performance.

#### 3.3.3 Ultraprecision Optical Quartz Glass Polishing Properties

Figure 10 shows the glass surface quality and MRR after polishing with our slurry prepared using the CeO_2_ sample synthesized at 6 h reaction time, 140 °C reaction temperature, and 0.06 mol/L cerium salt concentration. The average particle size of our synthesized CeO_2_ sample is 46.9 nm as shown in Figure 3c. In Figure 10a,c, the 2D and 3D images are displayed. Figure 10a shows that the surface flatness and uniformity of the glass surface are exceptionally high. Figure 10b shows the line-scan profile curve, and the peak-to-trough amplitude (the vertical distance between the highest and lowest points) of the glass surface is 0.77 nm, with peaks of 0.44 nm and troughs of 0.33 nm, which validates that the polishing solution made from our sample has further improved the surface quality of the glass. In Figure 10c, the nanoscale bumps on the glass surface can hardly be detected, truly achieving an ultrasmooth surface. The MRR at this time is 379 ± 0.58 nm/min, and the Ra is 0.11 ± 0.0013 nm, which is 0.03 nm lower than that used for semiconductors, and the roughness has been further improved. There is a highly promising application prospect in the field of ultraprecision optical instruments.

**Figure 10:**
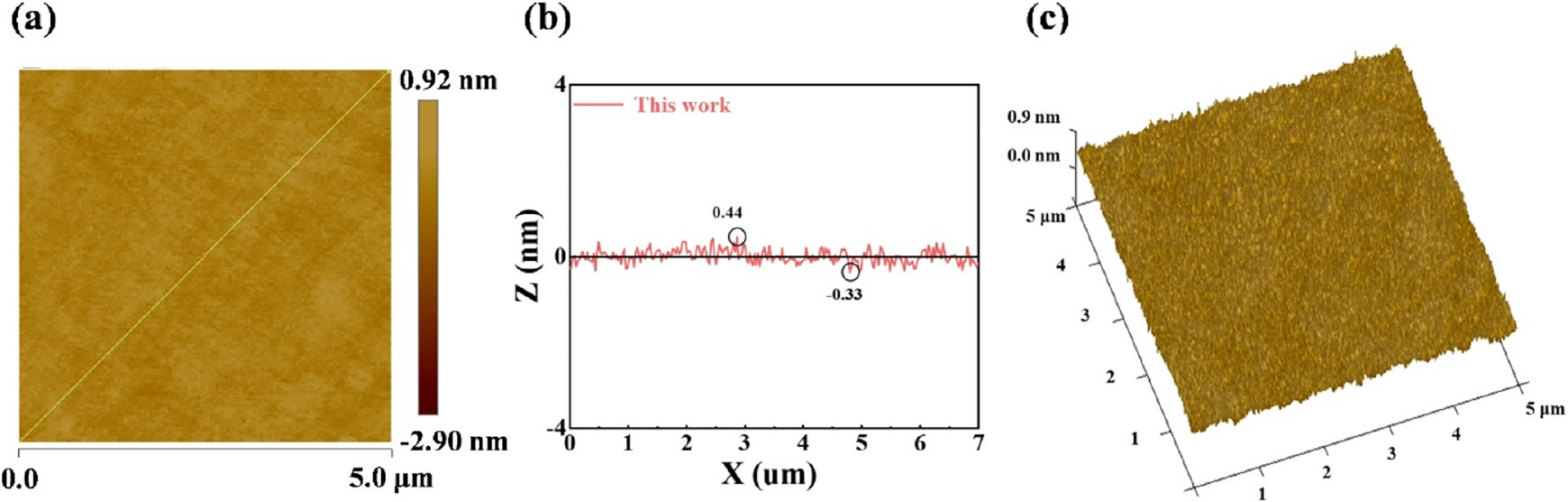
(a) 2D-AFM images, (b) line-scan profiles, and (c) 3D-AFM images of the surfaces polished using (46.9 nm) slurry.

## 4 Conclusions

The monodisperse CeO_2_ NPs with tunable sizes and uniform spherical morphology are successfully synthesized through a combination of the solvothermal reaction and in situ surface modification using PVP as a surfactant. By adjusting the reaction conditions, the particle size of CeO_2_ NPs can be precisely controlled within the range of 7.0 to 336.3 nm. The CeO_2_ NPs with the average sizes of 94.5 and 46.9 nm are prepared as polishing slurries, respectively, and tested for CMP performance on JGS1 quartz under identical conditions. Compared with the commercial CeO_2_ product, the as-prepared 94.5 nm CeO_2_ slurry achieved an MRR of 481 nm/min, approximately 125% higher than that of the commercial product, and a Ra of 0.14 nm, which is also much lower than that of 0.30 nm achieved by commercial CeO_2_. It is worth noting that an even lower Ra of 0.11 nm can be achieved by reducing the particle size of CeO_2_ slurry to 46.9 nm. These findings highlight the promising capabilities of CeO_2_ NPs for semiconductor and ultraprecision optical polishing, offering a promising pathway for achieving ultrasmooth surfaces through a simple and eco-friendly method.

## Supplementary Material



## Notes

Additional experimental details include graphs of the stability tests of this study’s polishing solutions and commercial polishing solutions (PDF)
