# Supplementary material for: Controllable Synthesis of Monodisperse CeO2 Nanoparticles with Tunable Sizes for Chemical-Mechanical Polishing
Source: ACS Omega. 2025 Aug 6;10(32):35678–88. doi: 10.1021/acsomega.5c02209 (PMC12368622; doi:10.1021/acsomega.5c02209)
Supplement: Supplementary file 1 [file ao5c02209_si_001.pdf]

Supporting Information

Controllable Synthesis of Monodisperse CeO_2 Nanoparticles with Tunable Sizes for Chemical-Mechanical Polishing

Yange Ma[a, b], Tuquan Qiu[a, b], Zhenxiang Zhao[a, b], Quan Zou[a, b], Bo Chen[a, b *], Qian Sun[a, b *], Shuangliang Zhao[a, b]

[a] *State Key Laboratory of Featured Metal Materials and Life-cycle Safety for Composite Structures and School of Chemistry and Chemical Engineering, Guangxi University, Nanning 530004, PR China.*

[b] *Guangxi Key Laboratory of Petrochemical Resource Processing and Process Intensification Technology, Guangxi University, Nanning 530004, PR China.*

*Corresponding authors:
Bo Chen (E-mail: chenbo@gxu.edu.cn)
Qian Sun (E-mail: sunqian@gxu.edu.cn)

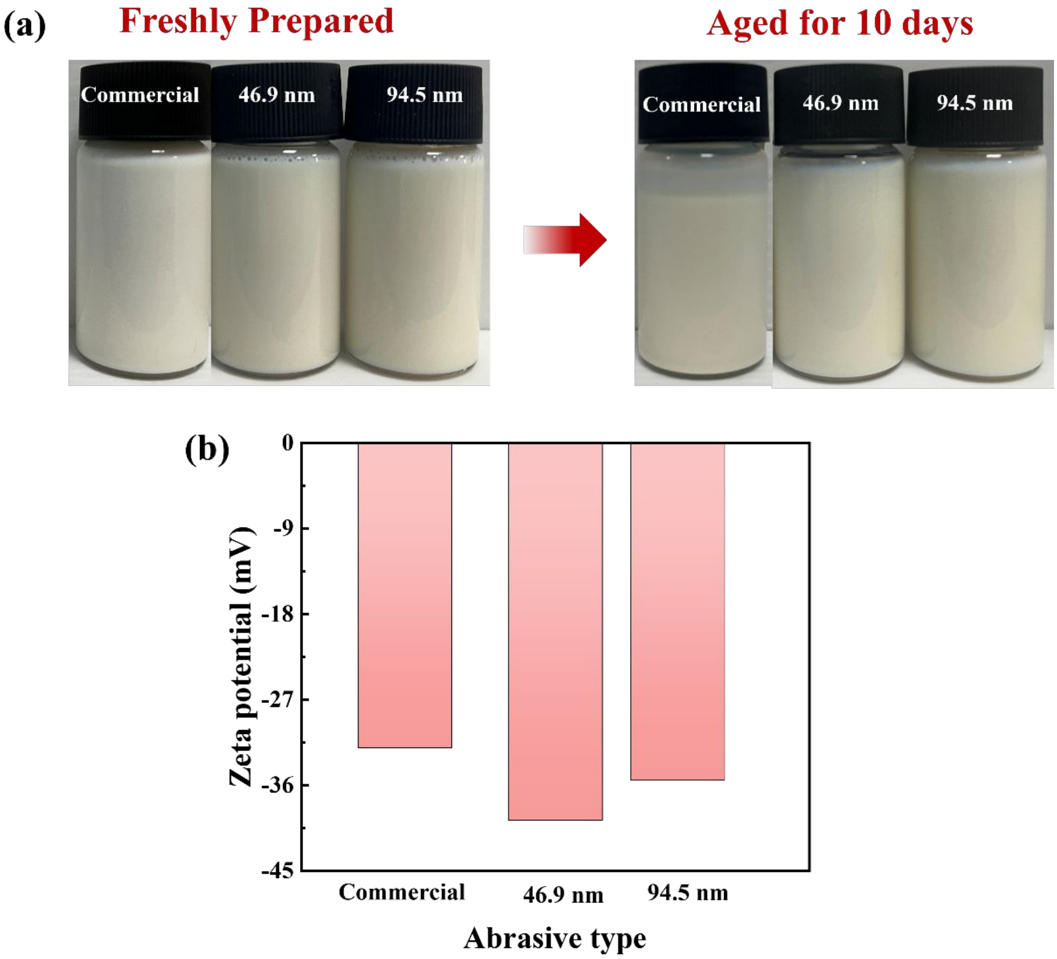

Figure S1. Stability comparison of different CeO_2 polishing slurries, using commercial CeO_2 and our modified CeO_2 with a diameter of 46.9 nm and 94.5 nm: (a) digital photographs, and (b) Zeta potential diagram.
